# Role of neuronal nitric oxide synthase (nNOS) at medulla in tachycardia induced by repeated administration of ethanol in conscious rats

**DOI:** 10.1186/s12929-018-0409-5

**Published:** 2018-01-31

**Authors:** Jiro Hasegawa Situmorang, Hsun-Hsun Lin, Hsuan Lo, Chih-Chia Lai

**Affiliations:** 1 Master and PhD Programs in Pharmacology and Toxicology, School of Medicine, Tzu Chi University, Hualien, Taiwan; 2 Department of Physiology, School of Medicine, Tzu Chi University, Hualien, Taiwan; 3 Department of Pharmacology, School of Medicine, Tzu Chi University, Hualien, Taiwan

**Keywords:** Ethanol, Heart rate, Tachycardia, nNOS, Ventral medulla, Autonomic nervous system

## Abstract

**Background:**

Intake of ethanol (alcohol) has been shown to influence cardiovascular function; the underlying brain mechanism remains unclear. Noting that nitric oxide (NO) system in the CNS is involved in the regulation of cardiovascular function, the present study examined the role of NO in medulla in ethanol-induced cardiovascular changes.

**Methods:**

Ethanol was administered by oral gavage at dose of 3.2 g/kg once every day for 8 consecutive days. Changes in blood pressure (BP) and heart rate (HR) in response to ethanol were measured by radiotelemetry method in freely moving female Sprague-Dawley rats. NO modulators were applied by intracerebroventricular (ICV) injection. The protein levels of nitric oxide synthase (NOS) and NO content in rostroventral medulla were measured by Western blot and nitrate/nitrite colorimetric assay kit, respectively.

**Results:**

Ethanol intake had little effects on basal BP and HR following 8 consecutive day treatments. A significant increase in HR but not BP following ethanol intake was observed at 6th and 8th, but not at 1st and 4th day treatments as compared with saline group. A decrease in the protein expression of neuronal NOS (nNOS) but not inducible NOS or endothelial NOS and a decline in the level of NO in the medulla 30 min after ethanol administration was observed at 8th day treatment. ICV treatment with NO donors attenuated ethanol-induced tachycardia effects at 8th day treatment. Ethanol produced significantly tachycardia responses when ICV nNOS inhibitors were given at 1st day treatment.

**Conclusion:**

Our results suggest that medulla nNOS/NO pathways play an important role in ethanol regulation of HR.

## Background

The detrimental effects of ethanol undoubtedly are the major concern worldwide. To date, ethanol is the most abused drugs in the world and it affects major organs such as brain and heart. The effects of ethanol consumption can vary according to the amount of intake, exposure duration in terms of acute and chronic intake, and type of ethanol beverages such as beer and wine. Chronic low and moderate ethanol intake has been shown to be cardiovascular protective while chronic high intake of ethanol leads to high risk of cardiovascular disease [1–3]. Ethanol also may increase heart rate in human [4, 5] or animals [6–8]. However, the mechanism underlying this phenomenon is not well characterized. It has been reported that the effect of ethanol on central nervous system (CNS), particularly nuclei in ventral region of medulla contain neurons that are responsible for cardiovascular regulation [9, 10], plays a role in mediating changes in cardiovascular function [11–16]. Thus, ethanol exposure may affect the function of the neurons in rostral ventrolateral medulla (RVLM) and the underlying cardiovascular regulation [17, 18].

Nitric oxide (NO) is a signaling molecule involved in neurotransmission within CNS as neuronal messenger [19]. NO is generated from amino acid L-arginine by the members of the NO synthase (NOS) family. There are three isoforms in the NOS family: neuronal NOS (nNOS), inducible NOS (iNOS) and endothelial NOS (eNOS) [20]. The three isoforms are localized differently in body’s organs. The constitute expression of iNOS isoform is low and induced under stress condition such as ischemia, trauma and inflammation [21, 22]. eNOS is mainly found in endothelial cells, vascular endothelium, and the smooth muscle. nNOS is a major isoform within the brain areas such as cerebral cortex, lateral dorsal, nucleus of solitary tract and cerebellum [23]. Previous studies have shown an increased vulnerability to ethanol-induced neuronal loss in the neocortex and hippocampus in neonatal mice genetically deficient for nNOS, suggesting a neuroprotective role of NO in ethanol intoxication [24]. In addition, central and peripheral NO and NOS have been demonstrated to participate in cardiovascular regulation [25–28] and may play a cardioprotective role [29, 30].

Acute or chronic ethanol intake affects NOS activity; ethanol regulation of NOS activity may vary in different brain cells [31]. It is not clear whether ethanol regulation of NO signals in the CNS is involved in the cardiovascular effects of ethanol. The present study was undertaken to test the hypothesis that repeated ethanol intake may impair the regulation of cardiovascular function and NOS/NO system in the ventral medulla. Using an in vivo model, we demonstrated that repeated administration of ethanol caused tachycardia, which may be consequential to reduction of nNOS protein levels and NO level in ventral medulla of brain by ethanol.

## Methods

### Animals

All animal care and experimental protocols were carried out in accordance with the guidelines of the Institutional Animal Care and Use Committee of Tzu Chi University. Female Sprague-Dawley rats, aged 11–12 weeks were purchased from commercial supplier (BioLASCO Co., LTD., Taipei, Taiwan). The rats were housed and maintained in controlled room at 23 °C ± 1 °C, with 50% ± 10% humidity and a 12-h light/dark cycle. After radiotelemetry transmitter surgery with or without intracerebroventricular cannula implantation, the rats were housed individually in separated cages.

### Determination of blood ethanol concentration

Blood ethanol concentrations were measured in another group of rats to avoid perturbing blood pressure and heart rate measurement. Prior to blood withdrawal, the rats were anesthetized using short-term inhalation anesthetic isoflurane (Panion & BF Biotech Inc., Taoyuan, Taiwan). The blood sample of 0.1 mL was withdrawn from the lateral tail vein using heparinized syringes at 30 min, 60 min, 120 min, and 180 min after oral gavage administration on day 1, day 3 and day 8. Blood ethanol levels were determined by an ethanol assay kit available commercially (BioAssay Systems, Hayward, CA); the rate of increase in absorbance at 580 nm was recorded with a microplate spectrophotometer (Epoch™, BioTek Instruments, Inc., Winooski, VT, USA).

### Radiotelemetry transmitter surgery and blood pressure measurement

Under anesthetized by intraperitoneal injection of Zoletil (50 mg/kg), rats were implanted with the transmitter (model PAC40, Data Sciences International). A 4 to 5-cm-long midline incision through the skin and wall of abdomen was made. The location of abdominal aorta was uncovered by using sterile cotton swab. To allow access to the abdominal aorta, the intestine was retracted by using a sterile cotton pad pre-soaked with normal saline. Four to five-cm-long suture was passed to each caudal to the left renal vein and anterior to the iliac bifurcation. Tension was applied to both sutures to allow temporary blood flow occlusion. Using a bended tip of 22-gauge needle, aorta 1–2 mm anterior to the iliac bifurcation was pierced and the catheter was inserted quickly upstream toward the heart. The aorta entry site was dried by using sterile cotton tip applicators and a small amount of Tissue adhesive (Vetbond, 3 M Animal Care Products, St Paul, MN, USA) was used to secure the catheter. Both occlusion sutures were slowly released and removed. The transmitter was placed inside the cavity and sutured to the abdominal wall. The abdominal wall and skin were closed individually. Rats were allowed for recovery at least 7 days before used in the following experiment. After recovery periods, rats were placed individually on a receiver and the transmitter was turned on or turned off by using a magnet. The blood pressure and heart rate data were analyzed using Dataquest A.R.T. System 2.2 software for Windows (Data Sciences International). The data was processed and counted per 60 min before treatment and per 10 min after administration of ethanol.

### Oral administration

Ethanol (3.2 g/kg, 40% *v*/v) or saline was given orally using 16-gauge 3-in.-long stainless steel needle (Cadence Science®, Cadence, Inc., Staunton, VA, USA). Before administration, the rats were restrained gently in upright position by grasping around the thorax to immobilize the head. The needle was inserted into the right side of the mouth and directed along the hard palate of the mouth to the back of the throat. Thereafter, the needle was passed into the esophagus until the base of the needle and ethanol or saline was delivered slowly.

### Intracerebroventricular cannula implantation

Procedures for intracerebroventricular (ICV) cannula implantation were similar to those described previously [32]. Briefly, under anesthetized by intraperitoneal injection of Zoletil (50 mg/kg), rats were placed prone in a David Kopf stereotaxic frame and the head was fixed by ear bars and incisor bar. Occipital hole was drilled to the desired position in relation to the bregma using the following stereotaxic coordinates: 1.5 mm lateral to the midline, 0.8 mm caudal to the bregma. A stainless steel guide cannula (23-gauge) was placed into the hole, 3.5 mm below the dorsal surface of the brain and secured with two stainless steel screws and dental cement. A removable stylet was used to close the cannula. A 27-gauge stainless steel micropipette was connected to Hamilton microsyringes (10 μL) and used for ICV administration; drugs (3 μL) were injected using a syringe pump at a rate of 10 μL/min. The drinking behavior caused by ICV angiotensin II (50 ng, 5 μL) 3 days after surgery was used to determine correct placement of the guide cannula [33]; the rats that started drinking within 3 min after the injection were used for the following experiments.

### Western blot analysis

The procedure for Western blot analysis of brain tissue was similar to that described in earlier studies [32, 34]. Rats were sacrificed 30 min after oral gavage of ethanol or saline. Brains were rapidly removed and soaked in ice-cold Krebs solution for 1 min. The brainstems were isolated from the brain and quickly frozen by cold spray (FREEZE 75; CRC Industry Europe NV, Zele, Belgium). Coronal section 0.5–1.5 mm rostral to the obex was prepared from the brainstem and the ventral part of the section was isolated. They were frozen in liquid nitrogen and stored at − 85 °C until used. About 10 mg of tissue was homogenized in 100 μL solution (0.32 M sucrose, 1 mM EDTA and 1 mTIU/ml aprotinin) with a homogenizer at speed of 10,000 rpm for 15 s. SDS was added to the sample to a final concentration of 0.1%, and 10 μg of protein was electrophoresed on 8% denaturing polyacrylamide gels. Separated proteins were transferred to PVDF transfer membrane and probed with primary antibody, rabbit anti-nNOS polyclonal antibody (1:1000, ab106417, Abcam, Cambridge, UK), rabbit anti-iNOS polyclonal antibody (1:1000, ab204017, Abcam, Cambridge, UK), and rabbit anti-eNOS polyclonal antibody (1:200, ab66127, Abcam, Cambridge, UK). Bound antibody was incubated with goat anti-rabbit secondary antibody (Bethyl, A120-101P, Bethyl Laboratories, Inc., Montgomery, TX, USA) conjugated to horseradish peroxidase which was reacted with Western Lighting® Plus-ECL Reagent (PerkinElmer, Waltham, MA, USA). The chemiluminescent signal was digitalized by UVP Biospectrum 810 (UVP, LLC, Upland, CA, USA) and the bands were analyzed with VisionWorks LS software for Windows (UVP, Upland). Protein concentrations were determined by BCA method (Sigma CO, St. Louis, MO, USA).

### Determination of total nitrate-nitrite

The rats were sacrificed 30 min following saline or ethanol administration and rostroventral medulla was carefully dissected from the brain stem as experiments for Western blot. Total nitrate-nitrite levels were measured by nitrate-nitrite colorimetric assay kit (Cayman Chemical, Ann Arbor, MI, USA) based on Griess method, in which a chromophore with a strong absorbance at 545 nm was formed. NO values obtained by this method represent the total amount of nitrate and nitrite expressed in pmol/mg tissue.

### Chemicals

Ethanol, NOC-18, and aprotinin were purchased from Sigma Co (St. Louis, Missouri, USA). NPLA was purchased from Tocris Cookson Ltd. (Bristol, UK). Zoletil® 50 was purchased from Virbac Taiwan Co. Ltd. (Taipei, Taiwan). Reagents for electrophoresis were purchased from Bio-Rad Laboratories (Richmond, CA).

### Statistical analysis

Data were presented as mean±S.E.M. and were plotted and analyzed statistically with GraphPad Prism version 6.03 for Windows, GraphPad Software (La Jolla California USA). The time course of percentage changes in BP and HR after administration of ethanol or NO modulators was analyzed using repeated measure one-way ANOVA followed by Dunnett’s post-test. The results of BP, HR and blood ethanol concentration among various days of treatment and the effects of NOS modulators on ethanol effects at different times after administration were analyzed using repeated measure two-way ANOVA followed by the Bonferroni post-test. The results of Western blot analysis and total nitrate-nitrite were analyzed by unpaired *t*-test (comparison of two groups). *P* < 0.05 was considered statistically significant.

## Results

### Effects of ethanol on mean arterial pressure and heart rate

**Fig. 1:**
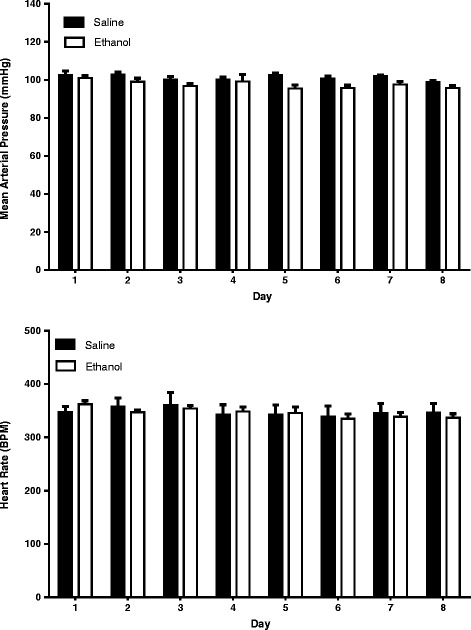
Bar graphs show basal MAP (top) and HR (bottom) before oral administration of 3.2 g/kg ethanol (*n* = 7) or saline (*n* = 7) at different days of treatment in freely moving rats. Ethanol (10 mL/kg, 40% (*v*/v)) or saline (10 mL/kg) was given by gavage once every day for 8 consecutive days. The values of MAP and HR represent an average value of 60 min recordings before ethanol or saline administration at each day of treatment

**Fig. 2:**
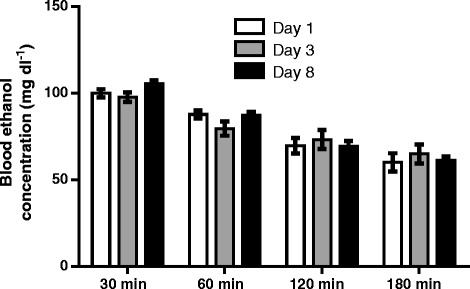
Bar graphic shows changes in blood ethanol concentrations at different times after administration of ethanol at day 1, 3, and 8 treatments. Ethanol (3.2 g/kg) was administered once every day for 8 consecutive days (*n* = 4)

**Fig. 3:**
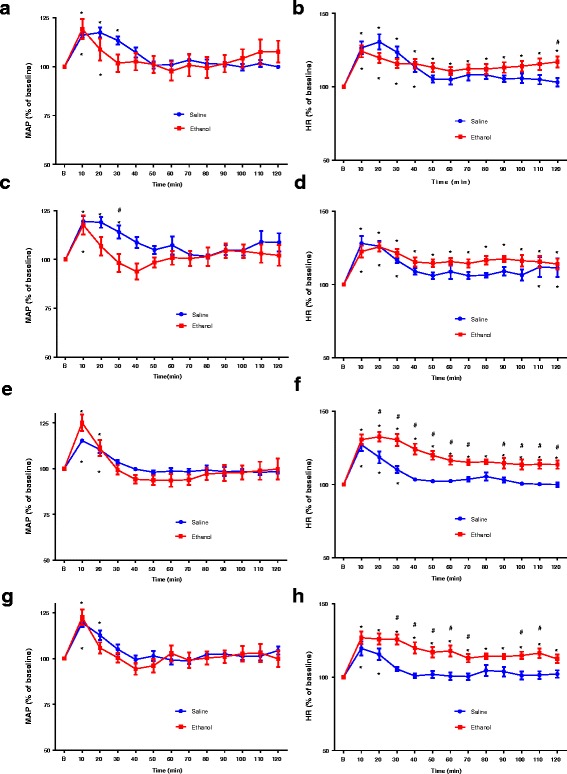
Line graph show the time course of changes in MAP and HR after oral gavage of saline or ethanol at 1st (**a**, **b**), 4th (**c**, **d**), 6th (**e**, **f**), 8th (**g**, **h**) day treatment. Ethanol (10 mL/kg, 40% (v/v)) or saline (10 mL/kg) were administered once every day for 8 consecutive days (*n* = 7 each). The average BP and HR 60 min before ethanol administration are taken as baseline (B) of 100%. *P < 0.05 compared with baseline (B) using repeated measure one-way ANOVA followed by Dunnett’s post-test. #*P* < 0.05 compared with saline group analyzed by repeated measure two-way ANOVA followed by Bonferroni post-test

The values of basal mean arterial pressure (MAP) and heart rate (HR) were measured 1-h prior to ethanol or saline administration. Basal MAP and HR of saline group (*n* = 7) and ethanol group (*n* = 7) were 102.6 ± 2.0 mmHg and 347.0 ± 11.1 bpm and 101.0 ± 1.4 mmHg and 362.1 ± 6.4 bpm, respectively, in free moving, conscious female rats on day 1 administration. The values of MAP and HR were measured for 8 consecutive days. Compared to saline group, basal MAP and HR of ethanol group were similar in the course of 8 days (Fig. 1). Changes in blood ethanol concentrations of ethanol at 30 min, 60 min, 120 min and 180 min following intake of ethanol at 1st, 3rd, and 8th days of treatment are illustrated in Fig. 2. Blood ethanol concentrations were about 100 mg/dL and 60 mg/dL at 30 min and 180 min, respectively, after oral gavage of 3.2 g/kg ethanol. There is no statistical difference in xethanol blood levels during the observation period among various days of treatment. Oral gavage of saline caused increases in MAP and HR at the early period (10–30 min) after administration. Oral gavage of ethanol caused increases in MAP at the early period (10–20 min) while increases in HR during the observation period (Fig. 3). In comparison to saline group, ethanol did not cause a significant change in MAP over time after exposure on day 1 (Fig. 3a), day 4 (Fig. 3c), day 6 (Fig. 3e), and day 8 (Fig. 3g) treatments. Similarly, ethanol caused no change in HR during ethanol exposure on day 1 (Fig. 3b) and day 4 (Fig. 3d) treatments. However, ethanol produced significantly increases in HR on day 6 (Fig. 3f) and on day 8 (Fig. 3h) treatments as compared with saline group. The tachycardia occurred at 20–30 min after ethanol intake and lasted over 60 min.

### Effects of ethanol on the protein level of NOS and total amount of nitrate-nitrite in rostroventral medulla

**Fig. 4:**
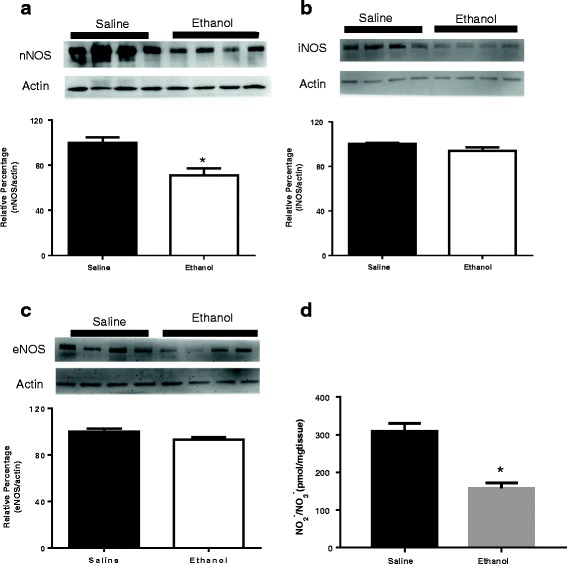
Bar graphs show Western blot analysis of the levels of nNOS (**a**), iNOS (**b**), eNOS (**c**) and changes in NOx (**d**) content in rostroventral medulla at 30 min after oral gavage of saline (10 mL/kg) or ethanol (3.2 g/kg) at day 8 treatment. Ethanol or saline was administered once every day for 8 consecutive days (*n* = 4 each). The ratio of different NOS to β-actin in saline group is taken as control of 100%. *P < 0.05 versus saline group analyzed using unpaired *t*-test

The changes in the levels of NOS expressions in the rostroventral part of medulla 30 min after oral administration of saline or ethanol were estimated by immunological staining of Western blots with antibody against nNOS, iNOS, and eNOS. The results showed that the protein level of nNOS was significantly decreased, without significant changes in the levels of iNOS and eNOS compared with saline group at 8th day treatment. Representative Western blots and percentage changes in the levels of nNOS, iNOS, and eNOS protein content in the rostroventral medulla are shown in Fig. 4a, b, and c, respectively. To determine changes in the NO content, the total amount of nitrate and nitrite (NOx) in the rostroventral medulla were measured following administration of ethanol. The NOx levels at 30 min after administration were significantly decreased at day 8 treatment of ethanol (158.26 ± 10.86 pmol/mg) compared with saline group (309.19 ± 16.42 pmol/mg) (Fig. 4d).

### Effects of NO modulators on ethanol regulation of HR

**Fig. 5:**
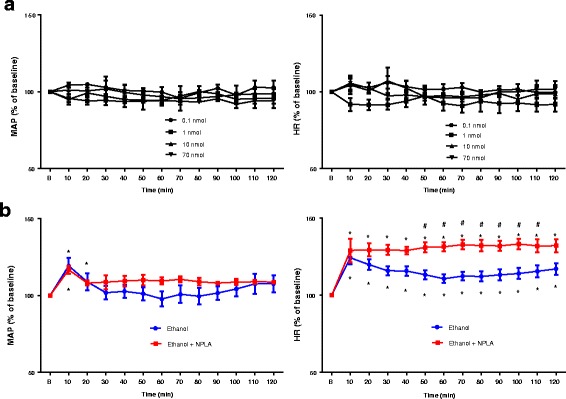
**a** Line graph shows the time course of percentage changes in MAP (left) and HR (right) after ICV injection of different doses of NPLA, a nNOS inhibitor (*n* = 4 each). The average BP and HR 60 min before NPLA injection are taken as baseline (**b**) of 100%. **b** Line graph show time course of percentage change in MAP (left) and HR (right) after administration of ethanol (3.2 g/kg) with or without ICV treatment with 10 nmol NPLA (*n* = 4) on day 1 treatment of ethanol. NPLA was given immediately after ethanol administration. The average BP and HR 60 min before ethanol administration are taken as baseline (**b**) of 100%. *P < 0.05 compared with baseline (**b**) using repeated measure one-way ANOVA followed by Dunnett’s post-test. #*P* < 0.05 compared with ethanol group analyzed by repeated measure two-way ANOVA followed by Bonferroni post-test

**Fig. 6:**
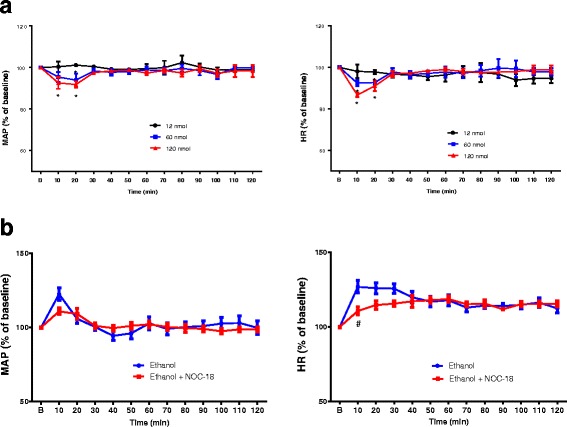
**a** Line graph shows the time course of percentage changes in MAP (left) and HR (right) after ICV injection of different doses of NOC-18, an 00000NO donor (*n* = 3 each). The average BP and HR 60 min before NOC-18 injection are taken as baseline (**b**) of 100%. *p < 0.05 compared with baseline using repeated measure one way ANOVA followed by Dunnett’s posttests. **b** Line graph show time course of percentage change in MAP (left) and HR (right) after administration of ethanol with or without ICV treatment with 12 nmol NOC-18 (*n* = 4) on day 8 treatment of ethanol. NOC-18 was given immediately after ethanol administration. The average BP and HR 60 min before ethanol administration are taken as baseline (**b**) of 100%. #*P* < 0.05 compared with no ICV treatment using repeated measure two-way ANOVA followed by Bonferroni post-test

ICV injection of NPLA (a nNOS inhibitor) alone in various dosages (up to 70 nmol) did not cause significant changes in MAP and HR over the observation period (Fig. 5a). However, ethanol produced a significant increases in HR but not MAP following ethanol intake in rats treated with ICV NPLA (10 nmol), which was applied immediately after oral gavage of ethanol, at day 1 ethanol treatment (Fig. 5b), suggesting that acute intake of ethanol may induce or potentiate the tachycardia effects while brain nNOS activity was impaired. ICV injection of a lower dose of NOC-18 (12 nmol, an NO donor) had little effects on MAP and HR. Higher doses of NOC-18 (60, 120 nmol) significantly reduced MAP and HR (Fig. 6a); the reduction lasted for about 20 min. Immediate ICV injection of low dose of NOC-18 (12 nmol) after ethanol administration reduced the tachycardia induced by ethanol at 8th day treatment; treatment with NOC-18 had no significant effects on BP responses (Fig. 6b).

## Discussion

In the present study, we showed that repeated daily administration of ethanol by oral gavage over 8 days did not significantly change the baseline BP and HR when compared to saline-treated rats. However, repeated ethanol intake caused a progressive increase in HR, but no significant changes in BP following ethanol administration. In addition, the protein level of nNOS and NO content decreased in rostroventral medulla after repeated administration of ethanol. We further showed that acute intake of ethanol produced tachycardia effects when brain nNOS activity was blocked by ICV nNOS inhibitors. ICV treatment with NO donor reduced the tachycardia induced by repeated administration of ethanol. Our results provide the first in vivo evidence that nNOS/NO system in the medulla of the brain participates in the regulation of HR by ethanol. The results imply that the central effects of ethanol play an important role in the mechanisms underlying ethanol regulation of cardiovascular function.

Central regulation of HR mainly relies on parasympathetic impulses from nucleus ambiguus (NA) and sympathetic impulses from RVLM. Both nuclei are located in ventral medulla oblongata. Parasympathetic stimulation has been known to slow down the HR; in contrast, sympathetic stimulation has been known to increase the HR. Ethanol has been repeatedly reported to increase HR [4–8] though the action mechanism is not well characterized. Some studies suggest that ethanol regulation of the activity of autonomic nuclei in the CNS plays a role in ethanol cardiovascular function. For instance, ethanol may inhibit pressor effects elicited by NMDA receptor stimulation in RVLM [17] or in contrast ethanol may induce pressor responses via the activation of NMDA receptors in the central nucleus of amygdala [11].

In the present study, ethanol did increase HR and BP following administration since day 1 treatment. Nevertheless, the increases in HR and BP existed in both ethanol group and saline group. This is reasonable because oral gavage administration of both ethanol and saline may impose stress on rats, leading to consequent increases in HR and BP. In line with our results, previous studies have shown that oral gavage caused a short-period increase in HR and BP [35]. On day 6 and 8 treatments, however, ethanol significantly increased HR after administration compared with saline group; the effects lasted for around 2-h observation period. The progressive increase in tachycardia responses to ethanol is not due to a baroreflex response because changes in MAP in both saline and ethanol groups are not statistically significant. The autonomic nervous system is crucial for regulation of BP and HR. Though BP and HR have close relationship each other, they are regulated by different mechanisms. Based on our findings, ethanol may have more effects on regulatory machinery of HR than that of BP. Our results showed a decrease in the protein levels and NO content in the rostroventral medulla during tachycardia responses after repeated daily ethanol intake. In addition, ICV application of NO modulators regulated ethanol-induced tachycardia effects. These results suggest that NO system in the autonomic nuclei in the CNS plays an important role in cardiovascular effects of ethanol. Moreover, ethanol-induced increases in HR after repeated administration were observed at blood concentrations of less than 100 mg/dL (0.1%) in the present study. Most heavy drinkers should reach this range of blood levels.

NO has been known as a key modulator in the brain and it has different effects on physiological function including cardiovascular function. Studies have shown that central endogenous and exogenous NO affects BP and HR [25–29]. The role of NO in regulation of BP and HR may vary depending on its location and/or under influence of the other substances. For instance, decreases in nNOS protein expression and NO levels by overexpression of cystathionine-β synthase in the RVLM elicited increases in BP and HR in spontaneously hypertensive rats [36]. The mechanisms of superoxide-induced sympathoexcitation in hypertensive rats may partly involve the reduction of NO-mediated GABA release in the RVLM [37]. Those studies imply that increases in NO production in the RVLM may lower BP and HR. On the other hand, studies showed that nNOS-mediated NO production exhibited excitatory tonic activity in the RVLM [38, 39]. The cardioprotection role of nNOS/NO system has been noted in several studies such as control of baroreceptor reflex sensitivity [40], and maintenance of hemodynamic response during brain activation [41]. Our data showed that nNOS protein expression in rostroventral medulla of ethanol-treated rats was reduced on the 8th day treatment compared to saline-treated rats while iNOS and eNOS protein expression remained the same. This implies that the development of tachycardia following administration of ethanol during the course of treatment may be due to the reduction of nNOS protein expression in the rostroventral medulla. In accord with the changes in nNOS protein level, the level of NO represented by total nitrate/nitrite in rostroventral medulla was decreased on day 8 treatment 30 min following administration of ethanol. Previous in vitro and in vivo studies have shown that acute exposure of ethanol had little effects on NOS and nNOS enzyme activity in several brain areas [42, 43]. Thus the decreased NO level is most likely due to reduction in the protein expression of nNOS. Further work would be required to clarify the mechanism underlying ethanol-induced decrease in protein level of NOS.

The present study was carried out in conscious rats. ICV administration of agents regulating nNOS/NO system is the best choice to further clarify the role of nNOS/NO system in HR changes upon repeated ethanol exposure pharmacologically. We examined the effect of ICV nNOS inhibitors on the cardiovascular function. The results showed that neither BP nor HR was affected by ICV nNOS inhibitors alone. Nevertheless, microinjection of nNOS inhibitors into RVLM has been reported to result in significant hypotension and bradycardia [38, 39]. It is likely that nNOS inhibitors after ICV administration are widely diffused in the brain and thus the cardiovascular responses may result from the summation of the activation or inactivation of neuronal activity in various autonomic nuclei. However, treatment with ICV nNOS inhibitors leads to a notable increase in HR following acute administration of ethanol. Central NO donor is known to reduce BP and HR [28, 44–46]. Similar to previous findings, our results showed that ICV NO donor can reduce BP and HR. These effects also can be seen when higher doses of NO donor were injected directly to RVLM [38]. Interestingly, our results showed attenuation of ethanol-tachycardia by ICV treatment with small dose of NO donor that has no effects on BP and HR though the attenuation only observed at early period after ethanol administration. The short-acting effect of NO donor may be due to rapid metabolism as our results showed that decreases in BP and HR following ICV administration of high doses of NO donor only lasted for a short period (10–20 min). By combining pharmacological and biochemical approaches, our results demonstrate the specific role of nNOS/NO system in the medulla in the tachycardia caused by repeated ethanol intake. Studies have documented the predominant role of NA, located in the ventral medulla, in regulation of HR [47]. Though our results are not able to indicate the exact site in the medulla due to the limitation of methodology (ICV administration of drugs), NA containing vagal cardioinhibitory neurons is the most likely site for ethanol regulation of HR because decreases in nNOS-mediated NO production may cause a reduction of neuronal activity in NA and a subsequent decrease in vagal tone, leading to tachycardia [48].

Ethanol concentration might be different in the brain among individual [49] and the effects of ethanol are highly correlated with its concentration in the blood. In our results, the blood ethanol concentration was not different between day 1 and day 8. This indicates that the effects of ethanol are not due to pharmacokinetic tolerance. The present study was carried out in female rats. It has been reported that ethanol reach peak concentration in the brain of female rats faster than male rats [50] and thus may cause worsen effect in female rats. In addition, it has been shown that acute ethanol-induced hypotensive effects in female rats are estrogen-dependent [51] . Additional experiments may be required to clarify the role of estrogen in HR responses during repeated administration of ethanol in female rats.

## Conclusion

In conclusion, repeated daily ethanol intake that leads to a blood ethanol level comparable to a heavy drinker over 8 days induced tachycardia and decreased nNOS/NO system in the rostroventral medulla. Decreasing brain nNOS activity enhanced the development of tachycardia responses; and restoration of brain NO content could reduce the tachycardia responses. Thus nNOS/NO system in the medulla may play an important role in mediating ethanol-induced tachycardia during repeated ethanol exposure.

## Abbreviations

BP: Blood pressure
CNS: Central nervous system
eNOS: Endothelial NOS
HR: Heart rate
ICV: Intracerebroventricular
iNOS: Inducible NOS
MAP: Mean arterial pressure
NA: Nucleus ambiguus
nNOS: Neuronal NOS
NO: Nitric oxide
NOS: Nitric oxide synthase
NOx: Nitrate and nitrite
RVLM: Rostral ventrolateral medulla

## References

[1] Vasdev S, Gill V, Singal PK (2006). Beneficial effect of low ethanol intake on the cardiovascular system: possible biochemical mechanisms. Vasc Health Risk Manag.

[2] Krenz M, Korthuis RJ (2012). Moderate ethanol ingestion and cardiovascular protection: from epidemiologic associations to cellular mechanisms. J Mol Cell Cardiol.

[3] Jones A, McMillan MR, Jones RW, Kowalik GT, Steeden JA, Pruessner JC, Taylor AM, Deanfield JE, Muthurangu V (2013). Habitual alcohol consumption is associated with lower cardiovascular stress responses--a novel explanation for the known cardiovascular benefits of alcohol?. Stress.

[4] Spaak J, Tomlinson G, CL MG, Soleas GJ, Morris BL, Picton P, Notarius CF, Floras JS (2010). Dose-related effects of red wine and alcohol on heart rate variability. Am J Physiol Heart Circ Physiol.

[5] van den Wildenberg E, Beckers M, van Lambaart F, Conrod PJ, Wiers RW (2006). Is the strength of implicit alcohol associations correlated with alcohol-induced heart-rate acceleration?. Alcohol Clin Exp Res.

[6] Ristuccia RC, Spear LP (2008). Adolescent and adult heart rate responses to self-administered ethanol. Alcohol Clin Exp Res.

[7] Saalfield J, Spear L (2014). Developmental differences in the effects of alcohol and stress on heart rate variability. Physiol Behav.

[8] Sparrow MG, Roggendorf H, Vogel WH (1987). Effect of ethanol on heart rate and blood pressure in nonstressed and stressed rats. Life Sci.

[9] Tolentino-Silva FP, Campos Junior RR, Russo AK, Cravo SL, Lopes OU (1997). Cardiorespiratory effects of L-glutamate microinjected into the rat ventral medulla. Respir Physiol.

[10] Keeler JR, Helke CJ (1985). Spinal cord substance P mediates bicuculline-induced activation of cardiovascular responses from the ventral medulla. J Auton Nerv Syst.

[11] Chapp AD, Gui L, Huber MJ, Liu J, Larson RA, Zhu J, Carter JR, Chen QH (2014). Sympathoexcitation and pressor responses induced by ethanol in the central nucleus of amygdala involves activation of NMDA receptors in rats. Am J Physiol Heart Circ Physiol.

[12] El-Mas MM, Abdel-Rahman AA (2014). Ser/thr phosphatases tonically attenuate the ERK-dependent pressor effect of ethanol in the rostral ventrolateral medulla in normotensive rats. Brain Res.

[13] El-Mas MM, Fan M, Abdel-Rahman AA (2013). Role of rostral ventrolateral medullary ERK/JNK/p38 MAPK signaling in the pressor effects of ethanol and its oxidative product acetaldehyde. Alcohol Clin Exp Res.

[14] Zhang X, Abdel-Rahman AA, Wooles WR (1989). Impairment of baroreceptor reflex control of heart rate but not sympathetic efferent discharge by central neuroadministration of ethanol. Hypertension.

[15] Varga K, Ethanol KG (1990). Inhibition of baroreflex bradycardia: role of brainstem GABA receptors. Br J Pharmacol.

[16] Appalsamy M, Robertson D, Mosqueda-Garcia R (1994). Inhibition by ethanol of the cardiovascular effects of glutamate in the nucleus of the solitary tract. Am J Med Sci.

[17] Lin HH, Chang SJ, Shie HJ, Lai CC (2006). Ethanol inhibition of NMDA-induced responses and acute tolerance to the inhibition in rat rostral ventrolateral medulla in vivo: involvement of cAMP-dependent protein kinases. Neuropharmacology.

[18] Lai CC, Chang MC, Lin HH (2004). Acute tolerance to ethanol inhibition of NMDA-induced responses in rat rostral ventrolateral medulla neurons. J Biomed Sci.

[19] Bredt DS, Snyder SH (1992). Nitric oxide, a novel neuronal messenger. Neuron.

[20] Guix FX, Uribesalgo I, Coma M, Munoz FJ (2005). The physiology and pathophysiology of nitric oxide in the brain. Prog Neurobiol.

[21] Malaviya R, Gow AJ, Francis M, Abramova EV, Laskin JD, Laskin DL (2015). Radiation-induced lung injury and inflammation in mice: role of inducible nitric oxide synthase and surfactant protein D. Toxicol Sci.

[22] Sunil VR, Shen J, Patel-Vayas K, Gow AJ, Laskin JD, Laskin DL (2012). Role of reactive nitrogen species generated via inducible nitric oxide synthase in vesicant-induced lung injury, inflammation and altered lung functioning. Toxicol Appl Pharmacol.

[23] Dawson TM, Snyder SH (1994). Gases as biological messengers: nitric oxide and carbon monoxide in the brain. J Neurosci.

[24] Bonthius DJ, RA MK, Koele L, Harb H, Kehrberg AH, Mahoney J, Karacay B, Pantazis NJ (2006). Severe alcohol-induced neuronal deficits in the hippocampus and neocortex of neonatal mice genetically deficient for neuronal nitric oxide synthase (nNOS). J Comp Neurol.

[25] Wang Y, Liu XF, Cornish KG, Zucker IH, Patel KP (2005). Effects of nNOS antisense in the paraventricular nucleus on blood pressure and heart rate in rats with heart failure. Am J Physiol Heart Circ Physiol.

[26] Carvalho TH, Lopes OU, Tolentino-Silva FR (2006). Baroreflex responses in neuronal nitric oxide synthase knoukout mice (nNOS). Auton Neurosci.

[27] Danson EJ, Mankia KS, Golding S, Dawson T, Everatt L, Cai S, Channon KM, Paterson DJ (2004). Impaired regulation of neuronal nitric oxide synthase and heart rate during exercise in mice lacking one nNOS allele. J Physiol.

[28] Chikada N, Imaki T, Seki T, Harada S, Nakajima K, Yoshimoto T, Naruse M, Demura H, Minami S, Takano K (2000). Distribution of c-fos mRNA in the brain following intracerebroventricular injection of nitric oxide (NO)-releasing compounds: possible role of NO in central cardiovascular regulation. J Neuroendocrinol.

[29] Burkard N, Williams T, Czolbe M, Blomer N, Panther F, Link M, Fraccarollo D, Widder JD, Hu K, Han H, Hofmann U, Frantz S, Nordbeck P, Bulla J, Schuh K, Ritter O (2010). Conditional overexpression of neuronal nitric oxide synthase is cardioprotective in ischemia/reperfusion. Circulation.

[30] Wang Y, Kodani E, Wang J, Zhang SX, Takano H, Tang XL, Bolli R (2004). Cardioprotection during the final stage of the late phase of ischemic preconditioning is mediated by neuronal NO synthase in concert with cyclooxygenase-2. Circ Res.

[31] Deng XS, Deitrich RA (2007). Ethanol metabolism and effects: nitric oxide and its interaction. Curr Clin Pharmacol.

[32] Lai CC, Kuo TI, Lin HH (2007). The role of protein kinase a in acute ethanol-induced neurobehavioral actions in rats. Anesth Analg.

[33] Fleegal MA, Sumners C (2003). Drinking behavior elicited by central injection of angiotensin II: roles for protein kinase C and Ca2+/calmodulin-dependent protein kinase II. Am J Physiol Regul Integr Comp Physiol.

[34] Keng NT, Lin HH, Lin HR, Hsieh WK, Lai CC (2012). Dual regulation by ethanol of the inhibitory effects of ketamine on spinal NMDA-induced pressor responses in rats. J Biomed Sci.

[35] Õkva K, Tamoševiciute E, Cižiute A, Pokk P, Rukšenas O (2006). Nevalainen T. Refinements For intragastric gavage in rats. Scand J Lab Anim Sci.

[36] Duan XC, Liu SY, Guo R, Xiao L, Xue HM, Guo Q, Jin S, Wu YM (2015). Cystathionine-beta-synthase gene transfer into rostral ventrolateral medulla exacerbates hypertension via nitric oxide in spontaneously hypertensive rats. Am J Hypertens.

[37] Shinohara K, Hirooka Y, Kishi T, Sunagawa K (2012). Reduction of nitric oxide-mediated gamma-amino butyric acid release in rostral ventrolateral medulla is involved in superoxide-induced Sympathoexcitation of hypertensive rats. Circ J.

[38] Chan SH, Wang LL, Wang SH, Chan JY (2001). Differential cardiovascular responses to blockade of nNOS or iNOS in rostral ventrolateral medulla of the rat. Br J Pharmacol.

[39] Martins-Pinge MC, Garcia MR, Zoccal DB, Crestani CC, Pinge-Filho P (2007). Differential influence of iNOS and nNOS inhibitors on rostral ventrolateral medullary mediated cardiovascular control in conscious rats. Auton Neurosci.

[40] Qadri F, Carretero OA, Scicli AG (1999). Centrally produced neuronal nitric oxide in the control of baroreceptor reflex sensitivity and blood pressure in normotensive and spontaneously hypertensive rats. Jpn J Pharmacol.

[41] Brozickova C, Otahal J (2013). Effect of an inhibitor of neuronal nitric oxide synthase 7-nitroindazole on cerebral hemodynamic response and brain excitability in urethane-anesthetized rats. Physiol Res.

[42] Ikeda M, Komiyama T, Sato I, Himi T, Murota S (1999). Neuronal nitric oxide synthase is resistant to ethanol. Life Sci.

[43] Brien JF, Reynolds JD, Cunningham MA, Parr AM, Waddock S, Kalisch BE (1995). Nitric oxide synthase activity in the hippocampus, frontal cerebral cortex, and cerebellum of the guinea pig: ontogeny and in vitro ethanol exposure. Alcohol (Fayetteville, NY).

[44] Nurminen ML, Vapaatalo H (1996). Effect of intracerebroventricular and intravenous administration of nitric oxide donors on blood pressure and heart rate in anaesthetized rats. Br J Pharmacol.

[45] Lin MT, Pan SP, Lin JH, Yang YL (1999). Central control of blood pressure by nitrergic mechanisms in organum vasculosum laminae terminalis of rat brain. Br J Pharmacol.

[46] Tseng CJ, Liu HY, Lin HC, Ger LP, Tung CS, Yen MH (1996). Cardiovascular effects of nitric oxide in the brain stem nuclei of rats. Hypertension.

[47] Stuesse SL, Fish SE (1984). Projections to the cardioinhibitory region of the nucleus ambiguus of rat. J Comp Neurol.

[48] Ruggeri P, Battaglia A, Ermirio R, Grossini E, Molinari C, Mary DA, Vacca G (2000). Role of nitric oxide in the control of the heart rate within the nucleus ambiguus of rats. Neuroreport.

[49] Quertemont E, Green HL, Grant KA (2003). Brain ethanol concentrations and ethanol discrimination in rats: effects of dose and time. Psychopharmacology.

[50] Robinson DL, Brunner LJ, Gonzales RA (2002). Effect of gender and estrous cycle on the pharmacokinetics of ethanol in the rat brain. Alcohol Clin Exp Res.

[51] El-Mas MM, Abdel-Rahman AA (1999). Estrogen-dependent Hypotensive effects of ethanol in conscious female rats. Alcohol Clin Exp Res.

